# Effect of Acetaminophen Alone and in Combination with Morphine and Tramadol on the Minimum Alveolar Concentration of Isoflurane in Rats

**DOI:** 10.1371/journal.pone.0143710

**Published:** 2015-11-25

**Authors:** Julio R. Chavez, José A. Ibancovichi, Pedro Sanchez-Aparicio, Carlos M. Acevedo-Arcique, Rafael Moran-Muñoz, Sergio Recillas-Morales

**Affiliations:** 1 Faculty of Veterinary Medicine, Department of Anesthesiology, Universidad Autónoma del Estado de México, El Cerrillo Piedras Blancas, Toluca, Estado de México, Mexico; 2 Faculty of Veterinary Medicine, Department of Pharmacology, Universidad Autónoma del Estado de México, El Cerrillo Piedras Blancas, Toluca, Estado de México, México; 3 Faculty of Veterinary Medicine, Universidad Autónoma de Yucatán, Department of Anesthesiology and Pharmacology, Merida, Yucatán, México; University of Bari, ITALY

## Abstract

**Background:**

It has been observed that acetaminophen potentiates the analgesic effect of morphine and tramadol in postoperative pain management. Its capacity as an analgesic drug or in combinations thereof to reduce the minimum alveolar concentration (MAC) of inhalational anesthetics represents an objective measure of this effect during general anesthesia. In this study, the effect of acetaminophen with and without morphine or tramadol was evaluated on the isoflurane MAC.

**Methods:**

Forty-eight male Wistar rats were anesthetized with isoflurane in oxygen. MAC_ISO_ was determined from alveolar gas samples at the time of tail clamping without the drug, after administering acetaminophen (300 mg/kg), morphine (3 mg/kg), tramadol (10 mg/kg), acetaminophen (300 mg/kg) + morphine (3 mg/kg), and acetaminophen (300 mg/kg) + tramadol (10 mg/kg).

**Results:**

The control and acetaminophen groups did not present statistically significant differences (*p =* 0.98). The values determined for MAC_ISO_ after treatment with acetaminophen + morphine, acetaminophen + tramadol, morphine, and tramadol were 0.98% ± 0.04%, 0.99% ± 0.009%, 0.97% ± 0.02%, and 0.99% ± 0.01%, respectively.

**Conclusions:**

The administration of acetaminophen did not reduce the MAC of isoflurane and did not potentiate the reduction in MAC_ISO_ by morphine and tramadol in rats, and therefore does not present a sparing effect of morphine or tramadol in rats anesthetized with isoflurane.

## Introduction

Acetaminophen, also known as paracetamol, has analgesic and antipyretic properties like NSAIDs. However, its classification is controversial, because unlike NSAIDs it has little anti-inflammatory activity, it does not induce side effects in the gastrointestinal tract and kidney, and it does not affect platelet function when applied at the recommended dose [1]. Acetaminophen is the drug of choice in patients where the administration of NSAIDs is contraindicated [2]. Although its clinical use began more than 100 years ago, its mechanism of action has not been fully determined. Indeed, acetaminophen has constantly been compared with NSAIDs, starting with Vane et al. [3] who demonstrated that the mechanism of action for aspirin-like drugs is the inhibition of prostaglandin synthesis. The mechanism by which acetaminophen exerts its action has been investigated in various research studies. Flower et al. [4] reported that the inhibition of brain cyclooxygenase is responsible for the antipyretic effect of paracetamol, generating the concept of a central mechanism of action. More recently, Chandrasekharan et al. [5] reported the existence of a variant of COX-1 in dogs. This enzyme, named COX-3 was identified in the central nervous system and was found to be selectively inhibited by antipyretic-analgesic drugs such as acetaminophen, suggesting a mechanism for pain and possibly fever reduction. Nevertheless, Kis et al. [6] recommended that these results should not be generalized to other species such as rodents and humans, and pointed out several methodological contradictions in the study. Currently, the existence of a third isozyme is the subject of debate [7]. A study by Hinz et al. [8] showed that acetaminophen is a selective COX-2 inhibitor in humans. In fact, there are reports of reduced inflammation in patients undergoing dental surgery [9]. At present, it is known that the mechanism of action of acetaminophen is complex because it involves the inhibition of cyclooxygenases both centrally and peripherally depending on the concentration of circulating peroxides. Therefore, some authors suggest classifying acetaminophen as an atypical NSAID [10]. At the same time, research has been conducted showing that acetaminophen is a prodrug, and indicating that the analgesic effect of acetaminophen is generated by the indirect activation of cannabinoid CB_1_ receptors [11,12]. Also, it has been shown that acetaminophen has an effect on the descending serotonergic pathway [13,14] and may interact with opioidergic systems [15,16] or nitric oxide pathways [17]. Overall, the mechanism of action of acetaminophen is currently not entirely clear.

Acetaminophen is used alone and in combination with other drugs—the administration of analgesic drugs with different mechanisms of action is often used to provide synergistic or additive effects, and in order to decrease the dose required to control pain and side effects [18]. Several studies have shown a morphine-sparing effect when morphine is combined with acetaminophen in the postoperative period [19,20]. Gomez de Segura et al. [21] demonstrated that aspirin potentiates the sparing effect of morphine on the MAC of isoflurane (MAC_ISO_) in rats. The opioid sparing effect of aspirin in perioperative management was demonstrated for the first time, using a reduction in the MAC of halogenated anesthetics as an objective measure to test the antinociceptive potency of analgesic drugs and their combinations in patients under general anesthesia [22,23]. To our knowledge, the benefit of combining acetaminophen and morphine in terms of reducing the minimum alveolar concentration (MAC) of isoflurane has not been reported, although acetaminophen is commonly used in the management of perioperative pain [24].

Another drug used in combination with acetaminophen is tramadol, an atypical opioid and synthetic analog of codeine [25,26]. These drugs have been clinically proven to be effective in the control of postoperative pain and in oncology patients [27,28] but there is no information about the effect of this combination on the MAC of inhaled anesthetics.

The aim of this study was evaluate the effect of acetaminophen on the reduction in MAC_ISO_ produced by morphine and tramadol in rats, and to test the hypothesis that acetaminophen synergistically potentiates the effect of morphine and tramadol on MAC_ISO_ reduction in rats.

## Materials and Methods

### Animals and husbandry

Forty-eight male Wistar rats, weighing 310 ± 20 g (290–330 g) were included in this experiment, which was approved by the Animal Research Ethics Committee for Animal Experimentation (protocol number 3492/2013CHT) of the Facultad de Medicina Veterinaria y Zootecnia, Universidad Autónoma del Estado de México. Rats were housed in groups of six in Plexiglas cages, with a 12 h light/12 h dark cycle (lights on at 07:00), with a relative humidity of 50–60% and ambient temperature 22 ± 2°C. The animals had free access to rodent food (Prolab® RMH 2500, USA) and water was provided ad libitum. Animals were allowed to acclimatize for at least one week before the studies took place. All the studies were performed during the morning (09:00–12:00). All animals were handled according to the guidelines set forth in the Guide for the Care and Use of Laboratory Animals.

### Anesthesia

Anesthesia induction was conducted by placing each rat in the induction chamber providing 5% isoflurane (Forane; Baxter Laboratories, USA) in a continuous oxygen flow of 5 L/min. Once the animal was anesthetized, tracheal intubation was performed with the animal positioned in dorsal recumbency using a 16-gauge catheter (Introcan; B-Braun, Brazil). A flexible, blunt-tip, wire guide was inserted into the trachea with an otoscope and used to direct the endotracheal catheter. Correct placement of the catheter was confirmed by CO_2_ infrared–absorption analysis (BeneView T5, mindray, Multi-gas offers, Nanshan, China). The catheter was then connected to a small T-piece breathing system with minimal dead space and a fresh gas flow of 1 L/min of oxygen. The isoflurane concentration was adjusted as necessary based on assessment of the palpebral reflex and hemodynamic responses during instrumentation. During the study, the rats were breathing spontaneously.

### Instrumentation and monitoring

The carotid artery was exposed via surgical cut-down and catheterized using a 24-gauge catheter (Introcan; B-Braun, Brazil); this was connected to a pressure transducer system for direct blood pressure monitoring and the collection of arterial blood to determine blood gases. Systolic, diastolic and mean arterial blood pressures (SAP, DAP and MAP, respectively) and heart rate (HR) were continuously monitored (BeneView T5, Mindray, Nanshan, China). For blood gas analysis, 0.3 mL of blood was obtained immediately before the first noxious stimulation and another one after determining the MAC (GEM Premier 3000; Instrumentation Laboratory, UK). The rectal temperature was maintained between 37°C and 38°C by means of a Convective Warming System (Equator®, SurgiVet®, Smiths Medical PM Inc., USA). The tail vein was catheterized using a 24-gauge catheter for the administration of drugs (Introcan; B-Braun, Brazil). Inspired ISO (Fi_Iso_), end-tidal (Fe_Iso_) concentrations, end-tidal carbon dioxide tension (PEtCO_2_) and respiratory rate (RR) were continuously measured with an infrared gas analyzer (BeneView T5, mindray, Multi-gas offers, Nanshan, China) by endotracheal gas sampling (60 mL/min) obtained by means of a catheter inserted through the endotracheal tube with the tip located at the level of the carina.

### MAC determination

Before the Control, MAC_ISO,_ and MAC_ISO + Treatment_ group determination, the inspired concentration of isoflurane was adjusted to 1.3%, which is a value close to the MAC of isoflurane (MAC_ISO_) reported in a study conducted by Criado et al. [29] in which, the MAC_ISO_ value determined in all rats was 1.29%. Once this concentration was achieved, it was maintained for 15 minutes, in order to achieve equilibrium between alveolar gas (end-tidal), arterial blood and the brain [22]. The MAC of isoflurane was determined by the tail clamp method described by Quasha et al. [22]. A painful noxious stimulus was applied with a hemostat clamped (20 cm Rochester Dean Hemostatic forceps) on the tail at a specific end-tidal concentration of each volatile agent. The tail was clamped to the first ratchet lock for 60 seconds. The tail was always stimulated proximally to the previous test site. A positive motor response was considered if jerking or twisting motion of the head, body, or movement of the extremities was observed. Negative responses included lack of movement of the head and limbs, muscle rigidity, shivering, tail movement, swallowing, and chewing.

If the response was negative, the concentration of isoflurane was decreased by 10% and maintained at this concentration for at least 15 minutes, before repeated application of the stimulus. When a positive response was elicited, the concentration of the volatile anesthetic was increased by 10%. The MAC_ISO_ was calculated as the mean value between the highest concentration that permitted movement in response to the stimulus and the lowest concentration that prevented such movement. In each rat, the MAC was evaluated in duplicate. The person assessing the response was blinded with respect to the drugs administered to each rat.

The isoflurane MAC values were corrected to sea level by use of the formula (barometric pressure of location /760 mmHg) x obtained MAC value. The mean barometric pressure was obtained from the official city meteorological station for the altitude at which the experiment was performed (2,680 m above sea level) and was 556 mmHg. At the end of each experiment, the animals were euthanized with pentobarbital given intravenously (Anestesal, Pfizer, México) to animals deeply anaesthetized with the inhalant agent.

### Experimental design

The 48 rats were randomly assigned to one of six groups (n = 8) using a random number generator (Excel 2007, Microsoft Office). Morphine 3 mg kg^-1^ IV (Graten, PiSA, México) was administered and the MAC determined (morphine group, n = 8). The acetaminophen group (n = 8) received 300 mg kg^-1^ IV of acetaminophen (Tempra IV, Bristol-Myers Squibb, Italy), the tramadol group (n = 8) received 10 mg kg^-1^ IV of tramadol (tramadol, AMSA, México) the acetaminophen + morphine group (n = 8) received 300 mg kg^-1^ IV of acetaminophen + 3 mg kg^-1^ IV of morphine and the acetaminophen + tramadol group (n = 8) received 300 mg kg^-1^ IV of acetaminophen + 10 mg kg^-1^ IV of tramadol. All drugs were administered into the tail vein within a period of 3–5 minutes to reduce cardiovascular and respiratory effects. The MAC of isoflurane was determined without prior drug administration (Control MAC_ISO_ group, n = 8). Forty-five minutes later the inspired concentration of isoflurane was adjusted to 1.3%. The MAC_ISO + Treatment_ determination was initiated 45 minutes after the administration of drugs and after the inspired concentration of isoflurane had been adjusted to 1.3%.

### Statistical analysis

Statistical analysis was performed using Prism 6 (GraphPad Software, Inc., USA). The Shapiro-Wilk test was used for the assessment of data normality. Data are reported as mean ± standard deviation (SD). Analysis of variance was performed and post hoc comparison of the groups was performed using the Holm-Sidak test. Values were considered statistically different when *p*< 0.05.

## Results

The time from the adjustment of the inspired concentration of isoflurane to 1.3% until MAC determination was 96 ± 10 min in the Control MAC_ISO_ group, the application of four stimuli being required. The time from the administration of drugs and the adjustment of the inspired concentration of isoflurane to 1.3% until MAC determination was 118 ± 1.4 min in the MAC_ISO + Treatment_ group, the application of six stimuli being required.

The mean ± SD Control MAC_ISO_ group value determined was 1.32% ± 0.06%. After acetaminophen administration the MAC_ISO + Treatment_ value was 1.31% ± 0.05%; this was not significantly different to the Control MAC_ISO_ group (*p* = 0.98). Morphine and tramadol were found to be equipotent, and decreased the MAC_ISO_ to 0.97% ± 0.02% and 0.99% ± 0.01%, respectively. These values were not significantly different from each other, but they were both significantly different from the Control MAC_ISO_ group (*p* = 0.0001). During the administration of morphine and tramadol, a decrease in arterial pressure and transient bradypnea was observed.

The administration of acetaminophen + morphine and acetaminophen + tramadol reduced the MAC_ISO_ to a level similar to that observed when only morphine or tramadol was administered. The values are shown in the Table 1 (S1 Table). The blood gas values, which were within normal parameters are shown in Table 2 (S2 Table).

**Table 1:** Effect of acetaminophen alone and in combination with morphine and tramadol on the minimum alveolar concentration of isoflurane in rats.

Group	MAC	% MAC reduction	P-value
Control MAC_ISO_ group	1.32 ± 0.06	-	-
MAC_ISO_+Acetaminophen	1.31 ± 0.05	0.76	0.98
MAC_ISO_+Tramadol	0.99 ± 0.01*	25	< 0.0001
MAC_ISO_+Morphine	0.97 ± 0.02*	26.5	< 0.0001
MAC_ISO_+Acetaminophen/Tramadol	0.99 ± 0.009*	25	< 0.0001
MAC_ISO_+Acetaminophen/Morphine	0.98 ± 0.04*	25.7	< 0.0001

*Statistically significant compared to the control group and the acetaminophen group (p < 0.05).

**Table 2:** Blood gas, bicarbonate and lactate values obtained when determining the MAC in the different treatment groups.

Variable	Control MAC_ISO_ group	Acetaminophen	Tramadol	Morphine	Acetaminophen Tramadol	Acetaminophen Morphine
pH	7.3 ± 0.02	7.3 ± 0.005	7.3 ± 0.01	7.3 ± 0.005	7.3 ± 0.01	7.3 ± 0.01
PaO_2_ mmHg	289. 3 ± 15	284.9 ± 7	270.4 ± 3	279.4 ± 10	285.9 ± 14	286.8 ± 13
PaCO_2_ mmHg	37.89 ± 0.15	40.09 ± 1	38.06 ± 2	37.06 ± 0.87	39.09 ± 3	40.09 ± 4
HCO_3_ mmHg	25.31 ± 0.31	24.43 ± 0.44	25.06 ± 0.37	23.06 ± 0.11	24.43 ± 0.42	23.43 ± 0.16
Lactate mmol/L	1.23 ± 0.05	1.35 ± 0.009	1.13 ± 0.008	1.13 ± 0.005	1.35 ± 0.007	1.35 ± 0.01

## Discussion

For the evaluation of analgesic efficacy resulting from the coadministration of NSAIDs and opioids, visual scales have been used, but these evaluations have been carried out in the postsurgical period [19,20,24]. The method used to evaluate the analgesic efficacy of a drug or combinations during general anesthesia assess the ability of drugs to reduce the MAC of inhalational anesthetics [22,23], which is defined as the end-tidal pressure of inhalant anesthetics necessary to avoid movement in 50% of individuals exposed to a supramaximal noxious stimulus [30].

This study shows the interaction in terms of MAC between acetaminophen, morphine and an atypical opioid, tramadol [25] in rats anesthetized with isoflurane.

In the present study, the Control MAC_ISO_ was similar to the isoflurane MAC reported by Criado et al. [29] who reported a value of 1.29% ± 0.08%, and the value reported by Wolff et al. [31] of 1.38% ± 0.05%. Similarly, the values determined for the MAC_ISO_ after morphine and tramadol were similar to those reported previously [31].

In the present experiment, we did not observe any interaction between acetaminophen and morphine or tramadol, which contrasts with the results reported by Benito et al. [32], where it was found that acetaminophen at the same doses used in this study potentiated the sevoflurane MAC reduction produced by remifentanil in rats.

There have been various studies on MAC sparing effects of different analgesic drugs [33,34,35,36]. However, the type of interaction that occurs between opioids and NSAIDs in terms of the reduction in the MAC of inhalational anesthetics has not shown consistent results. Vaughan et al. [37] identified a mechanism by which the inhibition of cyclooxygenase (COX) by NSAIDs potentiates the action of μ-receptor agonists in rats, by inhibiting GABAergic synaptic transmission in the opioid receptor-rich midbrain region periaqueductal grey area. One year later, it was shown that this synergistic effect is generated by the inhibition of COX-1 and not of COX-2 [38]. However, one study which evaluated the effect of selective COX-2 NSAIDs, such as meloxicam and carprofen in dogs [39], reported an additive effect in reducing the MAC of sevoflurane caused by butorphanol, a synthetic opioid which exerts its effects mainly at κ-opioid receptors. On the other hand, Santos et al. [40] reported a lack of interaction between meloxicam and morphine in reducing the isoflurane MAC in rats. Likewise, it has been reported that a COX-1 preferential flunixin, meglumine, does not potentiate the effect of morphine in reducing the isoflurane MAC [41]. This is in contrast to the potentiating effect of aspirin on morphine in reducing the isoflurane MAC reported by Gomez de Segura et al. [21].

In light of this information, it is possible that the type of interaction between NSAIDs and inhalational anesthetics is influenced by the type of halogenated drug. The difference between our study and that by Benito et al. [32] was that in our study, the effect of acetaminophen was evaluated in the MAC of isoflurane and not of sevoflurane and the difference in the halogenated anesthetic used may be the factor which caused the different effects observed. Similarly, the mechanisms by which NSAIDs potentiate the effect of opioids in terms of MAC do not correspond with the selectivity of NSAIDs for the different types of cyclooxygenase.

All these data suggest that the mechanism of action by which NSAIDs generate the potentiation of the effect of opiates on the MAC of inhalational anesthetics is variable and dependent on species susceptibility, the type of opioid. and other mechanisms by which these drugs may interact. Also, considering that NSAIDs are a large group of drugs with different mechanisms of action, caution should be employed in generalizing the type of interactions they can generate when combined with opiates.

## Conclusions

This study shows that acetaminophen does not reduce the MAC of isoflurane and does not potentiate the effect of morphine and tramadol in reducing the minimum alveolar concentration of isoflurane in rats. Therefore, acetaminophen does not present a sparing effect for morphine or tramadol in rats anesthetized with isoflurane.

## Supporting Information

S1 Table MAC of each of the individuals. (DOCX)

S2 Table Blood gases, bicarbonate and lactate in each individual when determining the MAC in the different groups. (DOCX)
