# Supplementary material for: Effect of Acetaminophen Alone and in Combination with Morphine and Tramadol on the Minimum Alveolar Concentration of Isoflurane in Rats
Source: PLoS One. 2015 Nov 25;10(11):e0143710. doi: 10.1371/journal.pone.0143710 (PMC4659611; doi:10.1371/journal.pone.0143710)
Supplement: S1 Table — (DOCX) [file pone.0143710.s001.docx]

**S1 Table**. MAC of each of the individuals

| Control MAC_ISO_ | MAC _ISO_ + Acetaminophen | MAC _ISO_ + Tramadol | MAC _ISO_ + Morphine | MAC _ISO_ + Acetaminophen/Tramadol | MAC _ISO_ + Acetaminophen/Morphine |
| --- | --- | --- | --- | --- | --- |
| 1.28 | 1.35 | 0.98 | 0.95 | 1.00 | 0.95 |
| 1.42 | 1.20 | 0.98 | 0.98 | 0.98 | 0.98 |
| 1.35 | 1.35 | 0.98 | 0.95 | 0.98 | 0.95 |
| 1.35 | 1.35 | 0.98 | 0.98 | 1.00 | 0.98 |
| 1.20 | 1.28 | 1.00 | 0.95 | 1.00 | 0.95 |
| 1.31 | 1.31 | 1.00 | 0.98 | 1.00 | 0.98 |
| 1.31 | 1.35 | 1.00 | 1.00 | 1.00 | 1.10 |
| 1.35 | 1.31 | 1.00 | 1.00 | 1.00 | 1.00 |

| Number of values | Control MAC_ISO_ | MAC _ISO_ + Acetaminophen | MAC _ISO_ + Tramadol | MAC _ISO_ + Morphine | MAC _ISO_ + Acetaminophen/Tramadol | MAC _ISO_ + Acetaminophen/Morphine |
| --- | --- | --- | --- | --- | --- | --- |
|  |  |  |  |  |  |  |
| Minimum | 1.200 | 1.200 | 0.9800 | 0.9500 | 0.9800 | 0.9500 |
| 25% Percentile | 1.288 | 1.288 | 0.9800 | 0.9500 | 0.9850 | 0.9500 |
| Median | 1.330 | 1.330 | 0.9900 | 0.9800 | 1.000 | 0.9800 |
| 75% Percentile | 1.350 | 1.350 | 1.000 | 0.9950 | 1.000 | 0.9950 |
| Maximum | 1.420 | 1.350 | 1.000 | 1.000 | 1.000 | 1.100 |
|  |  |  |  |  |  |  |
| Mean | 1.321 | 1.313 | 0.9900 | 0.9738 | 0.9950 | 0.9863 |
| Std. Deviation | 0.06424 | 0.05258 | 0.01069 | 0.02134 | 0.009258 | 0.04955 |
| Std. Error of Mean | 0.02271 | 0.01859 | 0.003780 | 0.007545 | 0.003273 | 0.01752 |
|  |  |  |  |  |  |  |
| Lower 95% CI | 1.268 | 1.269 | 0.9811 | 0.9559 | 0.9873 | 0.9448 |
| Upper 95% CI | 1.375 | 1.356 | 0.9989 | 0.9916 | 1.003 | 1.028 |
