# Supplementary material for: Effect of Acetaminophen Alone and in Combination with Morphine and Tramadol on the Minimum Alveolar Concentration of Isoflurane in Rats
Source: PLoS One. 2015 Nov 25;10(11):e0143710. doi: 10.1371/journal.pone.0143710 (PMC4659611; doi:10.1371/journal.pone.0143710)
Supplement: S2 Table — (DOCX) [file pone.0143710.s002.docx]

**S2 Table.** Blood gases, bicarbonate and lactate each individuals when determining the MAC in the different groups

pH

| Control | Acetaminophen | Tramadol | Morphine | Acetaminophen+  Tramadol | Acetaminophen+  Morphine |
| --- | --- | --- | --- | --- | --- |
|  |  |  |  |  |  |
| 7.33 | 7.33 | 7.30 | 7.33 | 7.30 | 7.30 |
| 7.33 | 7.33 | 7.30 | 7.33 | 7.30 | 7.30 |
| 7.37 | 7.33 | 7.30 | 7.33 | 7.30 | 7.30 |
| 7.37 | 7.34 | 7.33 | 7.34 | 7.33 | 7.33 |
| 7.37 | 7.34 | 7.33 | 7.34 | 7.33 | 7.33 |
| 7.33 | 7.33 | 7.30 | 7.33 | 7.30 | 7.30 |
| 7.33 | 7.34 | 7.33 | 7.33 | 7.33 | 7.33 |
| 7.37 | 7.34 | 7.30 | 7.34 | 7.30 | 7.30 |

| Number of values | Control | Acetaminophen | Tramadol | Morphine | Acetaminophen+  Tramadol | Acetaminophen+  Morphine |
| --- | --- | --- | --- | --- | --- | --- |
|  |  |  |  |  |  |  |
| Minimum | 7.330 | 7.330 | 7.300 | 7.330 | 7.300 | 7.300 |
| 25% Percentile | 7.330 | 7.330 | 7.300 | 7.330 | 7.300 | 7.300 |
| Median | 7.350 | 7.335 | 7.300 | 7.330 | 7.300 | 7.300 |
| 75% Percentile | 7.370 | 7.340 | 7.330 | 7.340 | 7.330 | 7.330 |
| Maximum | 7.370 | 7.340 | 7.330 | 7.340 | 7.330 | 7.330 |
|  |  |  |  |  |  |  |
| Mean | 7.350 | 7.335 | 7.311 | 7.334 | 7.311 | 7.311 |
| Std. Deviation | 0.02138 | 0.005345 | 0.01553 | 0.005176 | 0.01553 | 0.01553 |
| Std. Error of Mean | 0.007559 | 0.001890 | 0.005489 | 0.001830 | 0.005489 | 0.005489 |
|  |  |  |  |  |  |  |
| Lower 95% CI | 7.332 | 7.331 | 7.298 | 7.329 | 7.298 | 7.298 |
| Upper 95% CI | 7.368 | 7.339 | 7.324 | 7.338 | 7.324 | 7.324 |

PaO_2_

| Control | Acetaminophen | Tramadol | Morphine | Acetaminophen+  Tramadol | Acetaminophen+  Morphine |
| --- | --- | --- | --- | --- | --- |
| 270. | 270.9 | 269.2 | 270.2 | 296.0 | 270.0 |
| 271. | 280.0 | 269.0 | 281.0 | 289.8 | 286.0 |
| 302. | 285.0 | 270.0 | 290.0 | 290.0 | 294.7 |
| 291. | 296.0 | 268.0 | 295.0 | 299.0 | 304.0 |
| 302. | 289.0 | 272.0 | 270.4 | 285.0 | 299.0 |
| 272. | 290.0 | 268.0 | 288.0 | 285.9 | 277.0 |
| 302. | 287.0 | 269.0 | 270.4 | 270.9 | 294.0 |
| 304. | 281.0 | 278.0 | 270.3 | 270.3 | 270.0 |

|  | Control | Acetaminophen | Tramadol | Morphine | Acetaminophen+  Tramadol | Acetaminophen+  Morphine |
| --- | --- | --- | --- | --- | --- | --- |
|  |  |  |  |  |  |  |
| Minimum | 270.0 | 270.9 | 268.0 | 270.2 | 270.3 | 270.0 |
| 25% Percentile | 271.3 | 280.3 | 268.3 | 270.3 | 274.4 | 271.8 |
| Median | 296.5 | 286.0 | 269.1 | 275.7 | 287.9 | 290.0 |
| 75% Percentile | 302.0 | 289.8 | 271.5 | 289.5 | 294.5 | 297.9 |
| Maximum | 304.0 | 296.0 | 278.0 | 295.0 | 299.0 | 304.0 |
|  |  |  |  |  |  |  |
| Mean | 289.3 | 284.9 | 270.4 | 279.4 | 285.9 | 286.8 |
| Std. Deviation | 15.63 | 7.613 | 3.324 | 10.43 | 10.52 | 13.20 |
| Std. Error of Mean | 5.525 | 2.691 | 1.175 | 3.688 | 3.720 | 4.668 |
|  |  |  |  |  |  |  |
| Lower 95% CI | 276.2 | 278.5 | 267.6 | 270.7 | 277.1 | 275.8 |
| Upper 95% CI | 302.3 | 291.2 | 273.2 | 288.1 | 294.7 | 297.9 |

PaCO_2_

| Control | Acetaminophen | Tramadol | Morphine | Acetaminophen+  Tramadol | Acetaminophen+  Morphine |
| --- | --- | --- | --- | --- | --- |
| 37.7 | 39.9 | 35.0 | 36.0 | 37.0 | 37.0 |
| 37.7 | 39.4 | 36.3 | 36.1 | 36.9 | 35.0 |
| 38.0 | 39.5 | 37.2 | 37.9 | 39.8 | 38.0 |
| 38.0 | 41.1 | 40.0 | 37.7 | 45.0 | 44.0 |
| 38.0 | 43.0 | 44.0 | 38.0 | 42.4 | 46.0 |
| 37.7 | 38.1 | 38.0 | 36.1 | 35.9 | 37.8 |
| 38.0 | 38.7 | 36.3 | 37.0 | 37.7 | 37.9 |
| 38.0 | 41.0 | 37.7 | 37.7 | 38.0 | 45.0 |

| Number of values | Control | Acetaminophen | Tramadol | Morphine | Acetaminophen+  Tramadol | Acetaminophen+  Morphine |
| --- | --- | --- | --- | --- | --- | --- |
|  |  |  |  |  |  |  |
| Minimum | 37.70 | 38.10 | 35.00 | 36.00 | 35.90 | 35.00 |
| 25% Percentile | 37.70 | 38.88 | 36.30 | 36.10 | 36.93 | 37.20 |
| Median | 38.00 | 39.70 | 37.45 | 37.35 | 37.85 | 37.95 |
| 75% Percentile | 38.00 | 41.08 | 39.50 | 37.85 | 41.75 | 44.75 |
| Maximum | 38.00 | 43.00 | 44.00 | 38.00 | 45.00 | 46.00 |
|  |  |  |  |  |  |  |
| Mean | 37.89 | 40.09 | 38.06 | 37.06 | 39.09 | 40.09 |
| Std. Deviation | 0.1553 | 1.562 | 2.815 | 0.8766 | 3.136 | 4.212 |
| Std. Error of Mean | 0.05489 | 0.5521 | 0.9953 | 0.3099 | 1.109 | 1.489 |
|  |  |  |  |  |  |  |
| Lower 95% CI | 37.76 | 38.78 | 35.71 | 36.33 | 36.47 | 36.57 |
| Upper 95% CI | 38.02 | 41.39 | 40.42 | 37.80 | 41.71 | 43.61 |

HCO_3_

| Control | Acetaminophen | Tramadol | Morphine | Acetaminophen+  Tramadol | Acetaminophen+  Morphine |
| --- | --- | --- | --- | --- | --- |
| 25.3 | 24.5 | 24.9 | 23.0 | 24.8 | 23.2 |
| 25.7 | 24.6 | 25.9 | 23.2 | 24.7 | 23.8 |
| 24.9 | 23.8 | 25.0 | 23.0 | 24.8 | 23.6 |
| 25.3 | 25.0 | 24.8 | 23.3 | 23.6 | 22.5 |
| 24.8 | 24.0 | 25.0 | 23.0 | 24.6 | 23.4 |
| 25.6 | 24.6 | 24.8 | 23.0 | 24.4 | 23.2 |
| 25.5 | 24.0 | 25.3 | 23.0 | 24.5 | 23.8 |
| 25.4 | 24.9 | 24.8 | 23.0 | 24.0 | 23.9 |

| Number of values | Control | Acetaminophen | Tramadol | Morphine | Acetaminophen+  Tramadol | Acetaminophen+  Morphine |
| --- | --- | --- | --- | --- | --- | --- |
|  |  |  |  |  |  |  |
| Minimum | 24.80 | 23.80 | 24.80 | 23.00 | 23.60 | 22.50 |
| 25% Percentile | 25.00 | 24.00 | 24.80 | 23.00 | 24.10 | 23.20 |
| Median | 25.35 | 24.55 | 24.95 | 23.00 | 24.55 | 23.50 |
| 75% Percentile | 25.58 | 24.83 | 25.23 | 23.15 | 24.78 | 23.80 |
| Maximum | 25.70 | 25.00 | 25.90 | 23.30 | 24.80 | 23.90 |
|  |  |  |  |  |  |  |
| Mean | 25.31 | 24.43 | 25.06 | 23.06 | 24.43 | 23.43 |
| Std. Deviation | 0.3182 | 0.4432 | 0.3777 | 0.1188 | 0.4234 | 0.4621 |
| Std. Error of Mean | 0.1125 | 0.1567 | 0.1335 | 0.04199 | 0.1497 | 0.1634 |
|  |  |  |  |  |  |  |
| Lower 95% CI | 25.05 | 24.05 | 24.75 | 22.96 | 24.07 | 23.04 |
| Upper 95% CI | 25.58 | 24.80 | 25.38 | 23.16 | 24.78 | 23.81 |

Lactate

| Control | Acetaminophen | Tramadol | Morphine | Acetaminophen+  Tramadol | Acetaminophen+  Morphine |
| --- | --- | --- | --- | --- | --- |
| 1.17 | 1.35 | 1.13 | 1.13 | 1.35 | 1.34 |
| 1.20 | 1.35 | 1.13 | 1.14 | 1.34 | 1.34 |
| 1.28 | 1.34 | 1.15 | 1.13 | 1.34 | 1.35 |
| 1.28 | 1.37 | 1.13 | 1.13 | 1.35 | 1.36 |
| 1.28 | 1.35 | 1.13 | 1.12 | 1.36 | 1.34 |
| 1.17 | 1.34 | 1.13 | 1.13 | 1.35 | 1.36 |
| 1.28 | 1.35 | 1.12 | 1.13 | 1.35 | 1.38 |
| 1.18 | 1.35 | 1.14 | 1.13 | 1.36 | 1.34 |

| Number of values | Control | Acetaminophen | Tramadol | Morphine | Acetaminophen+  Tramadol | Acetaminophen+  Morphine |
| --- | --- | --- | --- | --- | --- | --- |
|  |  |  |  |  |  |  |
| Minimum | 1.170 | 1.340 | 1.120 | 1.120 | 1.340 | 1.340 |
| 25% Percentile | 1.173 | 1.343 | 1.130 | 1.130 | 1.343 | 1.340 |
| Median | 1.240 | 1.350 | 1.130 | 1.130 | 1.350 | 1.345 |
| 75% Percentile | 1.280 | 1.350 | 1.138 | 1.130 | 1.358 | 1.360 |
| Maximum | 1.280 | 1.370 | 1.150 | 1.140 | 1.360 | 1.380 |
|  |  |  |  |  |  |  |
| Mean | 1.230 | 1.350 | 1.133 | 1.130 | 1.350 | 1.351 |
| Std. Deviation | 0.05425 | 0.009258 | 0.008864 | 0.005345 | 0.007559 | 0.01458 |
| Std. Error of Mean | 0.01918 | 0.003273 | 0.003134 | 0.001890 | 0.002673 | 0.005154 |
|  |  |  |  |  |  |  |
| Lower 95% CI | 1.185 | 1.342 | 1.125 | 1.126 | 1.344 | 1.339 |
| Upper 95% CI | 1.275 | 1.358 | 1.140 | 1.134 | 1.356 | 1.363 |
